# Computational fluid dynamics analysis of the upper airway after rapid maxillary expansion: a case report

**DOI:** 10.1186/s40510-015-0085-x

**Published:** 2015-05-24

**Authors:** Ahmed Ghoneima, Sahar AlBarakati, Feifei Jiang, Katherine Kula, Tamer Wasfy

**Affiliations:** Department of Orthodontics and Oral Facial Genetics, Indiana University School Dentistry, 1121 West Michigan Street, Indianapolis, IN 46202 USA; Department of Orthodontics, Faculty of Dental Medicine, Al-Azhar University, Cairo, Egypt; Department of Pediatric Dentistry and Orthodontics, College of Dentistry, King Saud University, Riyadh, Saudi Arabia; Department of Mechanical Engineering, Indiana University School of Engineering, Indianapolis, IN USA

**Keywords:** Finite element analysis, Palatal expansion technique, Airway resistance

## Abstract

**Background:**

Assessment of the upper airway volume, morphology, and mechanics is of great importance for the orthodontic patient. We hypothesize that upper airway dimensions have significant effects on the dynamics of the airway flow and that both the dimensions and mechanics of the upper airway are greatly affected by orthodontic and orthopedic procedures such as rapid maxillary expansion (RME). The aim of the current study was to assess the effect of RME on the airway flow rate and pattern by comparing the fluid dynamics results of pre- and post-treatment finite element models.

**Methods:**

Customized pre- and post-treatment computational fluid dynamics models of the patient’s upper airway were built for comparison based on three-dimensional computed tomogram. The inhalation process was simulated using a constant volume flow rate for both models, and the wall was set to be rigid and stationary. Laminar and turbulent analyses were applied.

**Results:**

Comparisons between before and after RME airway volume measurements showed that increases were only detected in nasal cavity volume, nasopharynx volume, and the most constricted area of the airway. Pressure, velocity, and turbulent kinetic energy decreased after dental expansion for laminar and turbulent flow. Turbulent flow shows relatively larger velocity and pressure than laminar flow.

**Conclusions:**

RME showed positive effects that may help understand the key reasons behind relieving the symptom of breathing disorders in this patient. Turbulence occurs at both nasal and oropharynx areas, and it showed relatively larger pressure and velocity compared to laminar flow.

## Background

Rapid maxillary expansion (RME) is a dentofacial orthopedic procedure that is routinely used by dental clinicians and orthodontists to treat maxillary transverse discrepancies such as “posterior crossbites”; the same technique has been reported by some researchers to help expand the upper airway and improve breathing function in patients with nasal breathing disorders [1-3]. The main objective would be that correcting the existing posterior crossbite and widening of the maxillary dental arch is expected to reduce maxillary constriction and mouth breathing, thereby reliving the symptoms through decreasing the nasal resistance [1-5].

The human respiratory system can be divided into the upper and lower respiratory systems. The upper respiratory system consists of the nasal cavity, nasopharynx, oropharynx, and trachea. The shape and diameter of these passages determine the volume of air passing through them [6]. A patent airway and normal nasal breathing is considered crucial in the growth and development of craniofacial structures [7]. Reports have previously indicated that maxillary morphologic differences exist between individuals with normal airway systems and those with airway problems [6, 8, 9].

Computational fluid dynamics (CFD) is considered the most appropriate technique to simulate the internal flow dynamics of the upper airway. It also allows evaluation of the airflow in the nasal, nasopharynx, and oropharynx areas separately providing an accurate assessment tool [10, 11]. In addition, CFD also provides accurate simulation to the magnitudes of air pressure and velocity and thus more precise evaluation of the airway function [10]. Iwasaki et al. [11], in a CFD study, reported that in obstructive sleep apnea children, the pharyngeal airway pressure during inspiration decreases with the reduction of nasal resistance by RME [11].

Because of its impact on airway, maxillary expansion can potentially be used as a treatment option for airway constrictions. Multiple studies confirmed that RME increases airway widths and volumes [1-5]. However, there is lack of data on airway internal flow dynamics and patterns of airflow. The purpose of this study was to assess the effects of RME on the upper airway using CFD results of comparing pre- and post-treatment finite element models.

## Methods

Ethical approval was obtained from the university’s institutional review board committee (Indiana University), and informed consent was signed by the parents of the study subject. Pre- and post-treatment finite element models were constructed for a 9-year-old male with collapsed maxillary arch who suffer from improper nasal breathing. The patient was treated by RME as part of his comprehensive orthodontic treatment plan. He had no history of previous orthodontic or orthopedic treatment; no systemic diseases, craniofacial anomalies, or temporomandibular joint disorders; no tonsillectomy or adenoidectomy; no carious or periodontal lesions; and no metallic restorations.

The RME appliance used was a Hyrax appliance (Dentaurum, Ispringen, Germany), which included bands on the permanent first molars and first pre-molars. The patient was instructed to turn the expansion screw two times, twice daily (producing 0.8 mm of expansion daily) until the palatal cusps of the maxillary first molars contacted the buccal cusps of the mandibular first molars after about 2 weeks. Spiral computed tomography (CT) images were taken immediately before the RME and 3 months after the last activation of the appliance using the Xvision EX spiral machine (Toshiba Medical Systems, Otawara-Shi, Japan). Scans were made at 120 kV, 20 mA, and 0.4-mm voxel size, with a scanning time of 2 s per section and a 25-cm field of view. The window width was 2400 Hounsfield units. The machine’s perpendicular light beam was used to adjust the head position in all three planes and thus allow comparison of the pre- and post-treatment scans.

On the three-dimensional (3D) CT scans, the airway volume was measured at the nasal cavity, nasopharynx, oropharynx, hypopharynx, and the most constricted area of the airway using Dolphin imaging software version 11.0 (Dolphin Imaging, Chatsworth, CA, USA). The anatomic boundaries and airway outlines used are identified in Table 1. The same software was used for orienting the pre- and post-treatment 3D images. The midsagittal plane was adjusted on the skeletal midline of the face, the axial plane was lined up with the Frankfort horizontal plane, and the coronal plane was adjusted to pass through the level of the furcation point of the right maxillary first molar.

**Table 1:** Definition of airway boundaries

	Anterior boundary	Posterior boundary	Superior boundary	Inferior boundary
Nasal cavity	Line connecting the ANS to the tip of the nasal bone	Line extending from S to the PNS	Line connecting N to S	Line extending from ANS to PNS
Nasopharynx	Line extending from S to the PNS	Line extending from S to the tip of the odontoid process	Line extending from S to the tip of the odontoid process	Line extending from PNS to the tip of the odontoid process
Oropharynx	Line extending from PNS to the tip of the epiglottis	Line extending from the tip of the odontoid process to the posterior superior border of CV4	Line extending from PNS to the tip of the odontoid process	Line extending from the base of the epiglottis to the posterior superior border of CV4
Hypopharynx	Line extending from the base of the epiglottis to the inferior border of the symphysis	Line extending from the posterior–superior corner of CV4 to the posterior–inferior corner of CV4	Line extending from the base of the epiglottis to the posterior superior border of CV4	Line extending from the posterior–inferior corner of CV4 to the inferior border of the symphysis

*ANS* anterior nasal spine, *PNS* posterior nasal spine, *S* sella, *N* nasion, *CV* cervical vertebrae

In order to perform the fluid dynamics analysis of the upper airway, customized pre- and post-CFD finite element models of the upper airway of the patient were constructed. The inhalation process was simulated using a constant volume flow rate at the end of the airway for both models, and the wall was set to be rigid and stationary. Laminar and turbulent analyses were applied. Before and after RME models were evaluated using Mimics 13 (Materialise, Belgium) and ANSYS Workbench 12 (Canonsburg, PA, USA) softwares. The pressure, flow rate, and pattern of the upper airway were recorded and assessed. The software Mimics 13 (Materialise, Belgium) was used to process the scan images for upper airway segmentation. A threshold that best isolated the airway was set to segment the pre-treatment scan, and the same threshold was applied for the post-treatment scan. Manual modification was also applied after thresholding for detailed segmentation in the nasal area. The geometry of the airway was rebuilt after segmentation and then meshed in Mimics with a four-node tetrahedral element. The mesh was then exported to ANSYS Workbench 12 (Canonsburg, PA, USA). The geometrical changes were also measured after segmentation. Cervical vertebras (CV2 and CV5) were used as references to align pre- and post-treatment models. The most constricted area of the airway was selected and divided into ten cross-sections for comparison of finite element models.

The pressure at the nostrils was set to be zero at inlet, and the velocity at outlet was adjusted to match with the constant volume flow rate −300 mL/s [12]. Laminar and turbulent steady-state analyses (k-ε model) were applied for longitudinal comparison. Low level of under-relaxation factors from previous study was applied to stabilize the solution [13]. Pressure, velocity, geometrical change, and resistance of airway were compared to assess the effect of dental expansion. The resistance of airway is defined as:

$$ \mathrm{Resistance}\kern0.5em \mathrm{of}\kern0.5em \mathrm{airway}=\frac{\mathrm{Presssure}\kern0.5em \mathrm{difference}}{\mathrm{Flow}\kern0.5em \mathrm{rate}} $$

## Results

Comparisons between before and after RME airway volume measurements showed that increases were only detected in nasal cavity volume, nasopharynx volume, and the most constricted area of the airway (Table 2).

**Table 2:** Comparison between pre- and post-RME airway volume measurements

Parameters	Pre-RME	Post-RME	Change
Nasal cavity	32,746.8	36,585.1	3838.3
Nasopharynx	5499.6	6935.1	1435.5
Oropharynx	7680.5	7680.5	0
Hypopharynx	4557.8	4557.8	0
Most constricted area of the airway	112	127	15

For the element independence test, the element size was adjusted to be 3, 2, and 1 mm, and corresponding maximum velocity and pressure were recorded to show the independence. The result showed that 1-mm element size allowed accurate longitudinal comparison. The mesh comprised more than 158K nodes and 580K elements.

**Fig. 1:**
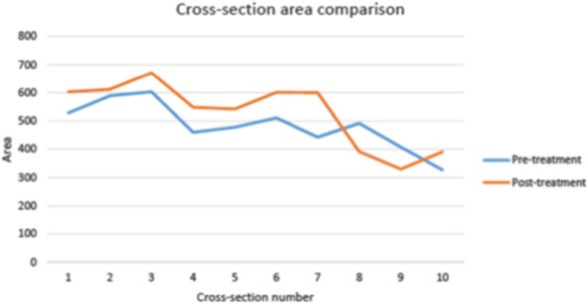
Cross-sectional area (mm^2^) comparison of the most constricted area of the upper airway

**Fig. 2:**
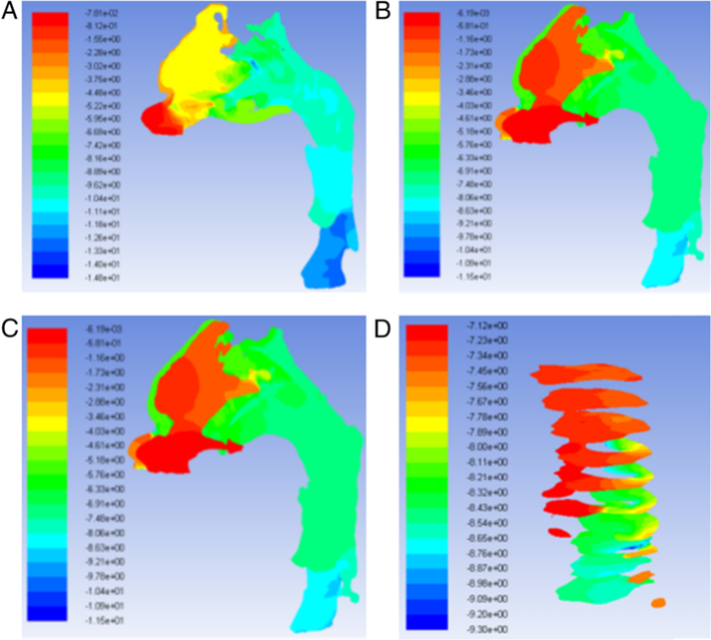
Pressure on the wall of laminar flow **a** pre-RME and **b** post-RME; pressure at cross-sections of laminar flow **c** pre-RME and **d** post-RME (Pa)

**Fig. 3:**
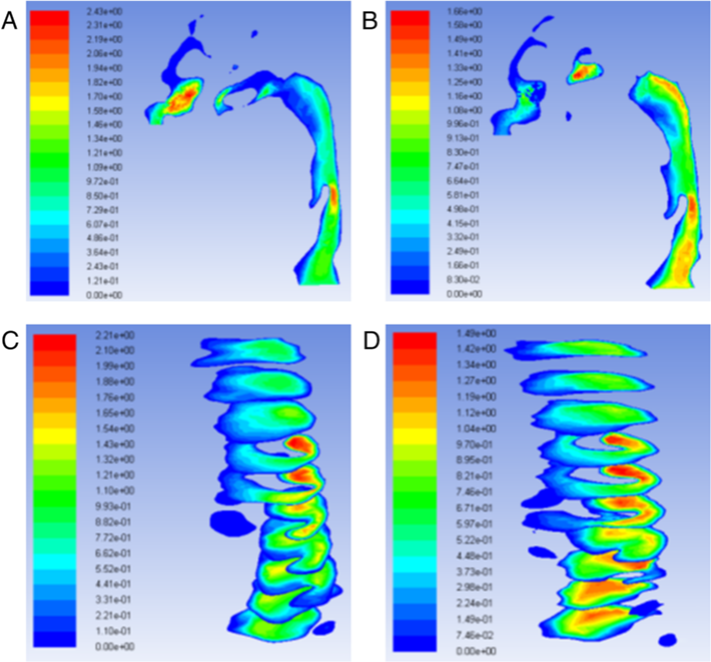
Velocity of laminar flow **a** pre-RME and **b** post-RME; velocity at cross-sections of laminar flow **c** pre-RME and **d** post-RME (m/s)

**Fig. 4:**
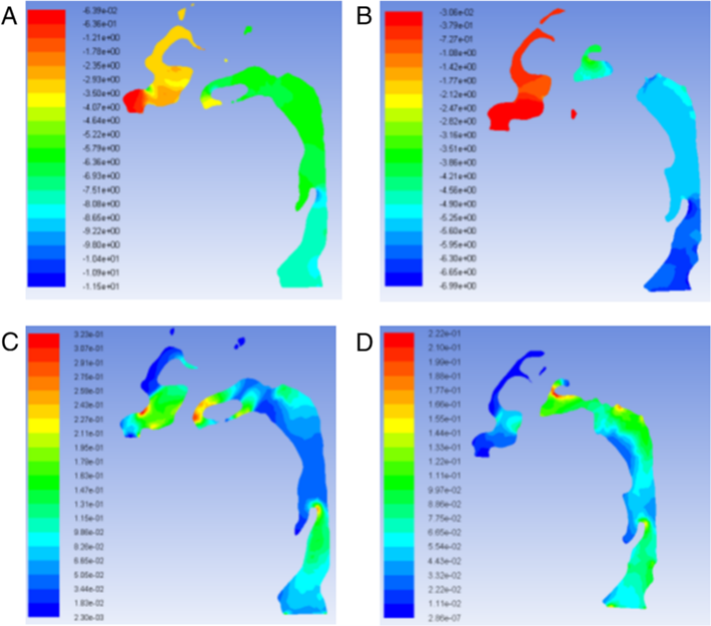
Pressure of turbulent flow **a** pre-RME and **b** post-RME (Pa); turbulence kinetic energy **c** pre-RME and **d** post-RME (m^2^/s^2^)

**Fig. 5:**
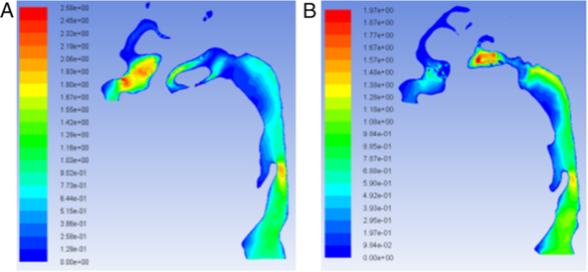
Velocity of turbulent flow **a** pre-RME and **b** post-RME (m/s)

**Fig. 6:**
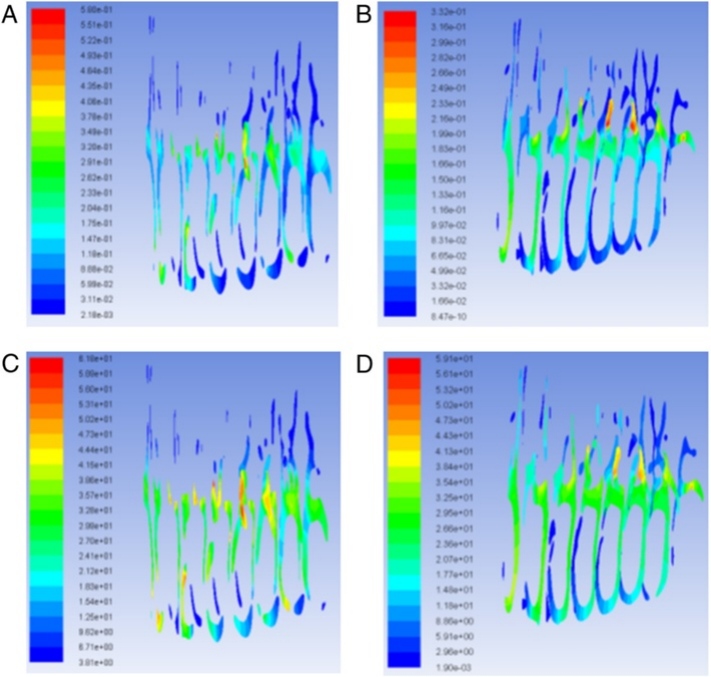
Turbulence kinetic energy at nasal cross-sections **a** pre-RME and **b** post-RME (m^2^/s^2^); turbulent intensity at nasal cross-sections **c** pre-RME and **d** post-RME (%)

The geometry and cross-sectional area comparison of the most constricted area of the upper airway is presented in Fig. 1. The CFD results comparison of laminar flow analysis are shown in Figs. 2 and 3. Resistance of airway between the first and last cross-section was 6.3 Pa·s/L for pre-treatment and 2.3 Pa·s/L for post-treatment. The CFD results comparison of turbulent flow analysis are shown in Figs. 4, 5, and 6.

**Fig. 7:**
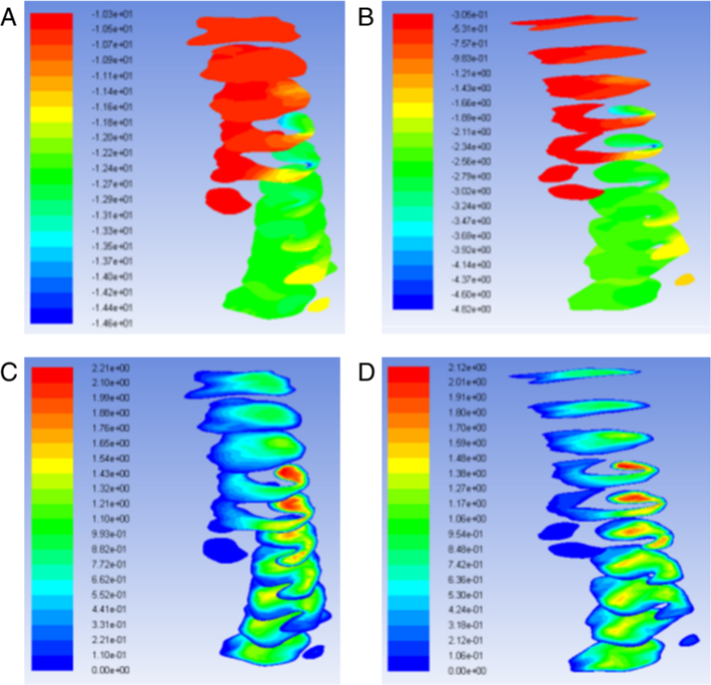
Pressure at cross-sections of laminar flow **a** integral model and **b** partial model (Pa); velocity at cross-sections of laminar flow **c** integral model and **d** partial model (m/s)

The pressure and velocity were compared at the cross-sections located in the same position. Pressure, velocity, and turbulent kinetic energy decreased after RME for laminar and turbulent flow, but they were similar for the integral and partial models. The pressure difference changed from 4.3 to 4.5 Pa, and the maximum velocity changed from 2.2 to 2.1 m/s. The variations were less than 5 %. Turbulent flow showed relatively larger velocity and pressure than laminar flow (Fig. 7).

## Discussion

Evaluation of the airway is critical for those patients who suffer from breathing problems and airway disorders. RME is widely used by clinicians to treat maxillary transverse discrepancies and is sometimes associated with improvement in the breathing patterns in patients with airway disorders. Literatures have reported the impact of RME on dental and skeletal structures, tongue posture, nasal morphology, and airway volume [14-17]. However, there is lack of data on airway internal flow dynamics and patterns of airflow in relation to RME. It is important to understand the effect of RME and increasing the upper airway volume in relation to the airflow dynamics in order to explore the exact mechanisms involved in relieving the symptoms associated with breathing disorders. The purpose of this study was to assess the effects of RME on the upper airway using CFD results of comparing pre- and post-treatment finite element models.

CFD is a convenient, reliable, and non-invasive evaluating tool for simulating the internal flow dynamics of the upper airway [18-21]. In the present study, CFD was used to provide information about the internal flow dynamics and measure the pressure, velocity, and turbulent kinetic energy of the upper airway of a male patient with collapsed maxilla. Results were compared between before and after RME treatment of the same patient. Three-dimensional evaluation of the airway volume was performed after dividing the airway passage into the nasal cavity, nasopharynx, oropharynx, hypopharynx, and the most constricted area of the airway. The results showed that increases were only detected in nasal cavity volume, nasopharynx volume, and the most constricted area of the airway after RME.

CFD results indicated that the geometry of airway showed some changes that were especially located at the nasal area. For laminar flow, the pressure dropped from −14.6 to −9.3 Pa after RME. The velocity dropped from 2.2 to 1.5 m/s. The resistance of airway was reduced from 6.3 to 2.3 Pa·s/L between the first and last cross-sections. Most importantly, CFD assessment of airway showed increase in geometry of the most constricted area of the airway. Comparison of laminar and turbulent flow analysis before and after RME indicated that when air flowed at low velocity and through a wider structure, pressure decreased and thus air appeared to flow in a more straight line “laminar flow.” Turbulence occurs at both nasal and oropharynx areas. Turbulent flow shows relatively larger pressure and velocity compared to laminar flow.

The pressure and velocity at the most constricted area of the airway decreased after RME for the laminar flow more than the turbulent flow although they both showed similar distribution patterns for the integral and partial models. The variations of both models were less than 5 %.

The results of the current study confirm the findings of Yu et al. [22] who investigated the effect of maxillomandibular advancement on two obstructive sleep apnea (OSA) patients’ upper airway flow dynamics using CFD. They indicated that the cross-sectional area of the narrowest part of the upper airway increased in all dimensions associated with decreased pressure gradient across the whole conduit during the passage of air. They stated that this was consistent with the clinical improvement and less breathing efforts reported for both cases. Similarly, Fan et al. [12], in their study, investigated the effect of surgical intervention on the size of the airway in an OSA patient using CFD. The results demonstrated significant pressure drop along the upper airway after the surgical procedure and suggested that this indicates decreasing collapsibility of the airway which will consequently improve the breathing function as well as the sleep quality.

Future studies are being planned with increased sample size and comparisons with gender- and age-matched control sample in order to overcome the current study sample size limitation.

## Conclusions

- Comparisons between before and after RME airway volume measurements showed that increases were only detected in nasal cavity volume, nasopharynx volume, and the most constricted area of the airway.
- Turbulence occurs at both nasal and oropharynx areas, and it showed relatively larger pressure and velocity compared to laminar flow.
- RME showed positive effects in terms of reduction of pressure, velocity, and resistance of airway in this patient. This may help understand the key mechanisms behind relieving the symptom of breathing disorders as a result of treatment with RME.

## References

[1] Haas AJ (1980). Long-term posttreatment evaluation of rapid palatal expansion. Angle Orthod.

[2] Bishara SE, Staley RN (1987). Maxillary expansion: clinical implications. Am J Orthod Dentofacial Orthop.

[3] Doruk C, Sökücü O, Sezer H, Canbay EI (2004). Evaluation of nasal airway resistance during rapid maxillary expansion using acoustic rhinometry. Europ J Orthod.

[4] Timms DJ (1984). The reduction of nasal airway resistance by rapid maxillary expansion and its effect on respiratory disease. J Laryngol Otol.

[5] Matsumoto MA, Itikawa CE, Valera FC, Faria G, Anselmo-Lima WT (2010). Long-term effects of rapid maxillary expansion on nasal area and nasal airway resistance. Am J Rhinol Allergy.

[6] Wong ML, Sandham A, Ang PK, Wong DC, Tan WC, Huggare J (2005). Craniofacial morphology, head posture, and nasal respiratory resistance in obstructive sleep apnoea: an inter-ethnic comparison. Eur J Orthod.

[7] Moss-Salentijn L, Melvin L (1997). Moss and the functional matrix. J Dent Res.

[8] Kilic N, Oktay H (2008). Effects of rapid maxillary expansion on nasal breathing and some naso-respiratory and breathing problems in growing children: a literature review. Int J Pediatr Otorhinolaryngol.

[9] Gungor AY, Turkkahraman H (2009). Effects of airway problems on maxillary growth: a review. Eur J Dent.

[10] Iwasaki T, Saitoh I, Takemoto Y, Inada E, Kanomi R, Hayasaki H (2012). Improvement of nasal airway ventilation after rapid maxillary expansion evaluated with computational fluid dynamics. Am J Orthod Dentofacial Orthop.

[11] Iwasaki T, Takemoto Y, Inada E, Sato H, Suga H, Saitoh I (2014). The effect of rapid maxillary expansion on pharyngeal airway pressure during inspiration evaluated using computational fluid dynamics. Int J Pediatr Otorhinolaryngol.

[12] Fan Y, Cheung LK, Chong MM, Chua HD, Chow KW, Liu CH (2011). Computational fluid dynamics analysis on the upper airways of obstructive sleep apnea using patient-specific models. IAENG INT J Comp Sci.

[13] Jeong SJ, Kim WS, Sung SJ (2007). Numerical investigation on the flow characteristics and aerodynamic force of the upper airway of patient with obstructive sleep apnea using computational fluid dynamics. Med Eng Phys.

[14] Kim YJ, Hong JS, Hwang YI, Park YH (2010). Three-dimensional analysis of pharyngeal airway in preadolescent children with different anteroposterior skeletal patterns. Am J Orthod Dentofacial Orthop.

[15] Chaconas SJ, Caputo AA (1982). Observation of orthopedic force distribution produced by maxillary orthodontic appliances. Am J Orthod.

[16] Starnbach H, Bayne D, Cleall J, Subtelny JD (1966). Facioskeletal and dental changes resulting from rapid maxillary expansion. Angle Orthod.

[17] Iwasaki T, Saitoh I, Takemoto Y, Inada E, Kakuno E, Kanomi R (2013). Tongue posture improvement and pharyngeal airway enlargement as secondary effects of rapid maxillary expansion: a cone-beam computed tomography study. Am J Orthod Dentofacial Orthop.

[18] De Backer JW, Vos WG, Gorle CD, Germonpre P, Partoens B, Wuyts FL (2008). Flow analyses in the lower airways: patient‐specific model and boundary conditions. Med Eng Phys.

[19] Xu C, Sin S, McDonough JM, Udupa JK, Guez A, Arens R (2006). Computational fluid dynamics modeling of the upper airway of children with obstructive sleep apnea syndrome in steady flow. J Biomech.

[20] Mihaescu M, Murugappan S, Gutmark E, Donnelly LF, Khosla S, Kalra M (2008). Computational fluid dynamics analysis of upper airway reconstructed from magnetic resonance imaging data. Ann Otol Rhinol Laryngol.

[21] Sung SJ, Jeong SJ, Yu YS, Hwang CJ, Pae EK (2006). Customized three‐dimensional computational fluid dynamics simulation of the upper airway of obstructive sleep apnea. Angle Orthod.

[22] Yu CC, Hsiao HD, Lee LC, Yao CM, Chen NH, Wang CJ (2009). Computational fluid dynamic study on obstructive sleep apnea syndrome treated with maxillomandibular advancement. J Craniofac Surg.

